# Supplementary material for: A comprehensive in silico investigation into the nsSNPs of Drd2 gene predicts significant functional consequences in dopamine signaling and pharmacotherapy
Source: Sci Rep. 2021 Dec 1;11:23212. doi: 10.1038/s41598-021-02715-z (PMC8636637; doi:10.1038/s41598-021-02715-z)
Supplement: Supplementary file 1 — Supplementary Information. [file 41598_2021_2715_MOESM1_ESM.pdf]

Supplementary Table S1. Distribution of STK11 intron, missense, coding synonymous, non-coding transcript variant and intron, 3'UTR, and 5'UTR SNPs.

SNP	Amount
Intron	15370 (93.40%)
Missense	238 (1.45%)
Synonymous	173 (1.05%)
Non-coding	139 (0.84%)
In-frame deletion	2 (0.01%)
Total	16456

Supplementary Table S2. Unanimous deleterious or damaging missense SNPs in the DRD2 protein predicted by 8 different tools.

nsSNP ID	Amino Acid Substitution	SIFT Score	PROVEAN Score	Polyphen 2 probability	SNAP 2 Score	PANTHER-PSEP preservation time	P-Mut Score	SNPs & GO probability	PhD-SNP score
rs752405585	Y37C	0	-4.11	1	40	750	0.8674	0.799	0.664
rs868401982	E62K	0	-3.15	1	82	1037	0.7875	0.578	0.642
rs1053323059	V74F	0.001	-4.63	1	44	750	0.5014	0.75	0.844
rs753530257	A77T	0	-3.72	1	79	1037	0.9043	0.726	0.83
rs199961459	A84T	0.001	-3.64	1	52	750	0.8055	0.627	0.595
rs1274176960	T119M	0	-5.7	1	84	1037	0.9179	0.736	0.855
rs148478679	C126W	0	-9.08	0.999	89	1037	0.9345	0.871	0.949
rs549053606	D131N	0.004	-4.74	0.958	80	1037	0.9305	0.75	0.862
rs779267865	R145C	0.008	-4.27	0.999	78	750	0.7467	0.777	0.862
rs199517364	R150H	0.001	-4.47	1	35	750	0.7933	0.683	0.798
rs754343100	R150C	0	-6.86	1	49	750	0.7057	0.761	0.874
rs773041317	G173R	0.005	-7.61	1	83	1037	0.867	0.803	0.879
rs987305741	R219C	0	-5.58	1	64	750	0.9345	0.638	0.779
rs890174542	E368D	0.002	-2.72	1	48	1037	0.6824	0.722	0.639
rs763380657	F389V	0	-5.81	1	74	1037	0.9345	0.729	0.889
rs773693505	C399R	0.001	-6.24	1	11	1037	0.9179	0.855	0.925
rs748707296	P404R	0.005	-3.03	0.996	14	750	0.8497	0.516	0.8
rs140938110	C443F	0	-6.13	1	71	1036	0.8657	0.727	0.715

Supplementary Table S3. Structural impact prediction of DRD2 high-risk pathogenic nsSNPs of DRD2 protein.

**Amino Acid Substitution**		**Y37C**	**E62K**	**V74F**	**A77T**	**A84T**	**T119M**	**C126W**	**D131N**	**R145C**
Mutpred prediction		Benign	Pathogenic	Pathogenic	Pathogenic	Pathogenic	Pathogenic	Pathogenic	Pathogenic	Pathogenic
Mupro stability prediction		Decrease	Decrease	Decrease	Decrease	Decrease	Increase	Decrease	Decrease	Decrease
NetSurfP prediction	native	Buried	Exposed	Buried	Buried	Exposed	Buried	Buried	Buried	Exposed
	mutant	Buried	Exposed	Buried	Buried	Buried	Buried	Buried	Buried	Exposed
mCSM Prediction		Destabilizing	Destabilizing	Destabilizing	Destabilizing	Destabilizing	Destabilizing	Destabilizing	Destabilizing	Destabilizing
SDM prediction		Stabilizing	Stabilizing	Destabilizing	Destabilizing	Destabilizing	Stabilizing	Stabilizing	Destabilizing	Destabilizing
DUET prediction		Destabilizing	Stabilizing	Destabilizing	Destabilizing	Destabilizing	Stabilizing	Destabilizing	Destabilizing	Destabilizing
Change in size		Decrease	Increase	Increase	Increase	Increase	Increase	Increase	Unchanged	Decrease
Change of charge		Unchanged	Negative > Positive	Unchanged	Unchanged	Unchanged	Unchanged	Unchanged	Negative > Neutral	Positive > Neutral
Change in hydrophobicity		Increase	Unchanged	Unchanged	Loss	Loss	Increase	Unchanged	Unchanged	Increase
Other impacts		None	None	None	None	None	None	The residue being involved in a cysteine bridge, the mutation will have a severe effect on the 3D-structure.	None	The residue being involved in a cysteine bridge, the mutation will have a severe effect on the 3D-structure.

Supplementary Table S3. Structural impact prediction of DRD2 high-risk pathogenic nsSNPs of DRD2 protein. (continued)

**Amino Acid Substitution**		**R150H**	**R150C**	**G173R**	**R219C**	**E368D**	**F389V**	**C399R**	**P404R**	**C443F**
Mutpred score		Pathogenic	Pathogenic	Pathogenic	Pathogenic	Pathogenic	Pathogenic	Pathogenic	Pathogenic	Pathogenic
Mupro stability prediction		Decrease	Decrease	Decrease	Decrease	Decrease	Decrease	Decrease	Decrease	Increase
NetSurfP prediction	native	Exposed	Exposed	Exposed	Exposed	Exposed	Buried	Exposed	Exposed	Exposed
	mutant	Exposed	Exposed	Exposed	Buried	Exposed	Exposed	Exposed	Exposed	Exposed
mCSM Prediction		Destabilizing	Destabilizing	Destabilizing	Destabilizing	Destabilizing	Destabilizing	Stabilizing	Destabilizing	Destabilizing
SDM prediction		Destabilizing	Destabilizing	Destabilizing	Destabilizing	Destabilizing	Stabilizing	Stabilizing	Destabilizing	Stabilizing
DUET prediction		Destabilizing	Destabilizing	Destabilizing	Destabilizing	Destabilizing	Destabilizing	Stabilizing	Stabilizing	Destabilizing
Change in size		Decrease	Decrease	Increase	Decrease	Decrease	Decrease	Increase	Increase	Increase
Change of charge		Positive > Neutral	Positive > Neutral	Neutral > Positive	Positive > Neutral	Unchanged	Unchanged	Positive > Neutral	Neutral > Positive	Unchanged
Change in hydrophobicity			Increase	Increase	Increase	Unchanged	Unchanged	Decrease	Decrease	Unchanged
Other impacts		None	The residue being involved in a cysteine bridge, the mutation will have a severe effect on the 3D-structure.	Exclusively the native glycine forms an unusual torsion angle. The mutation will force the local backbone into an incorrect conformation and will disturb the local structure.	The mutation will disturb interaction with secondary messenger Neurabin 2.	The wild-type forms a H bond with Gln 368 and a salt bridge with Lys 369. The mutation will disturb interaction with secondary messenger Neurabin 2.	The mutation being located within the agonist binding region, the resulting empty space in the core can disturb the functionality of the protein	The residue being involved in a cysteine bridge, the mutation will have a severe effect on the 3D-structure.	The mutation can disturb the special backbone conformation induced by the native proline residue.	None

Supplementary Table S4. Evolutionary conservation prediction of DRD2 protein using ConSurf and HOPE server

**Residue and Position**	**Conservation score**	**Prediction**	**HOPE conservation prediction**
C126	9	Highly conserved and buried (s)	Very conserved
R145	8	Very conserved and exposed (f)	Very conserved
R150	9	Highly conserved and exposed (f)	Very conserved
G173	9	Highly conserved and buried (s)	Very conserved
R219	9	Highly conserved and exposed (f)	Very conserved
E368	9	Highly conserved and exposed (f)	Very conserved
F389	9	Highly conserved and buried (s)	100% conserved
C399	9	Highly conserved and buried (s)	Very conserved
P404	7	Conserved and exposed	Not very conserved

Supplementary Table S5. Protein model verification by PROCHECK and ERRAT.

Mutation	Quality Parameters				
	PROCHECK Ramachandran plot				ERRAT quality factor
	Residues in most favoured regions (%)	Residues in additional allowed regions (%)	Residues in generously allowed regions (%)	Residues in disallowed regions (%)	
**Wild type**	91.1	6.7	1.1	1.1	78.7958
**C126W**	91.1	8.1	0	0.8	91.4787
**R145C**	90.1	9.1	0.3	0.5	84.6939
**R150C**	91.1	7.8	0.3	0.8	91.2281
**G173R**	89.8	7.8	1.6	0.8	94.7917
**R219C**	90.1	9.1	0.3	0.5	84.6939
**E368D**	90.3	7.5	1.1	1.1	88.2199
**F389V**	90.3	7.5	0.8	1.3	81.4136
**C399R**	90.3	8.6	0.3	0.8	86.9674

Supplementary Table S6. Interatomic interaction prediction of native DRD2 and mutant proteins.

Mutation	ΔΔG ENCoM	ΔΔS ENCoM	ΔΔG DynaMut
C126W	0.55	-0.688	1.229
R145C	-0.174	0.218	-0.512
R150C	0.403	-0.504	0.412
R219C	-0.34	0.425	-0.621
E368D	-0.267	0.334	-0.838
F389V	-0.655	0.819	-0.206
C399R	-0.501	0.626	0.054

Supplementary Table S7. Binding affinity (kcal/mol) prediction of ligands with native and mutant using PyRx.

**Ligand**	**Binding affinity (kcal/mol)**	
	**Wild type**	**Mutant (F389V)**
Dopamine	-5.8	-5.4
Risperidone	-8.5	-8.9

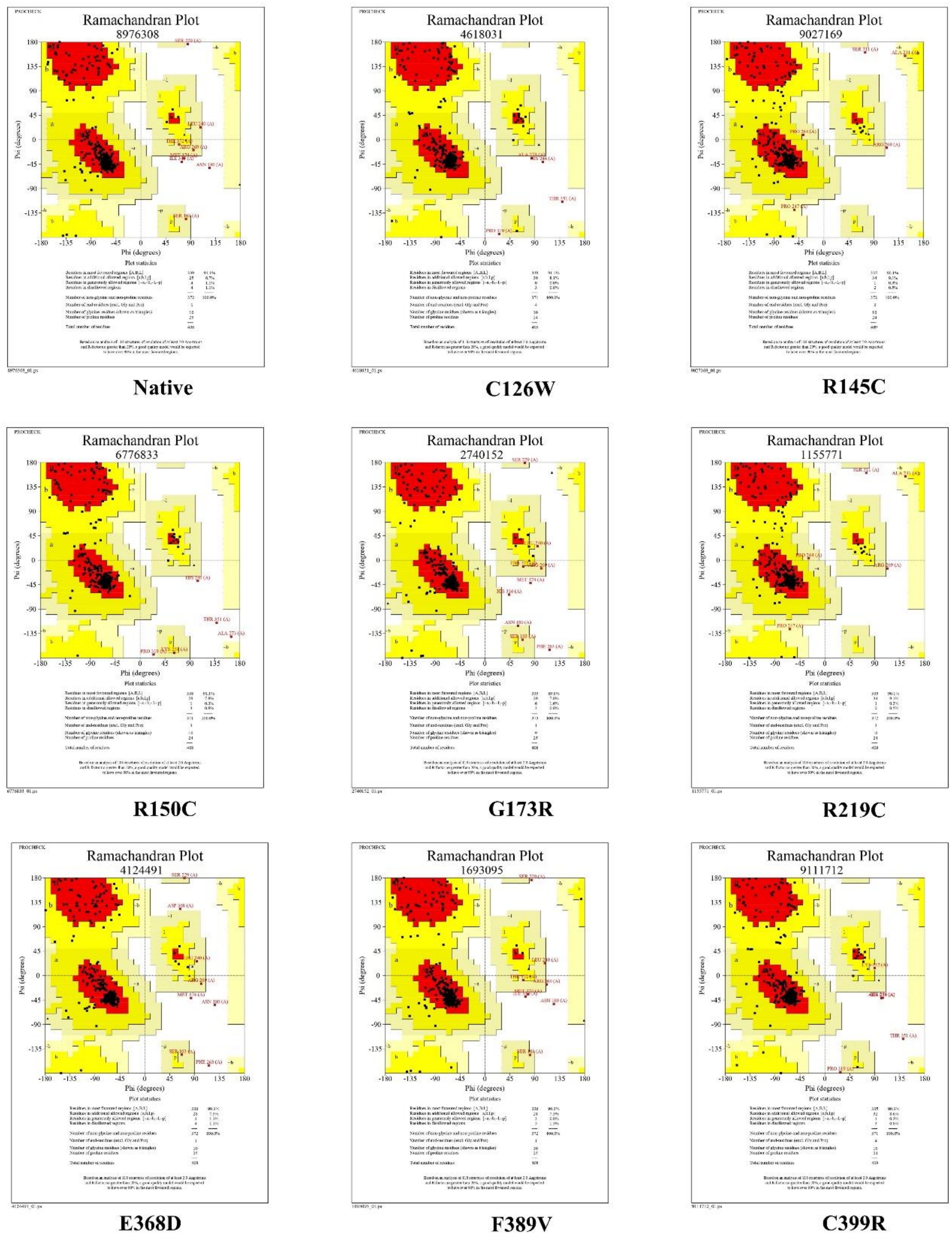

Supplementary Figure 1. Ramachandran plots of native and mutants derived from PROCHECK.
